# The necessity for surgeons to embrace transcatheter tricuspid valve therapy

**DOI:** 10.1016/j.xjse.2026.100143

**Published:** 2026-08-18

**Authors:** John N. Rachko, Ella Malynowski, Gilbert H.L. Tang

**Affiliations:** a Albert Dorman Honors College, New Jersey Institute of Technology, Newark, NJ; b Grace Church School, New York, NY; c Department of Cardiovascular Surgery and Medicine (Cardiology), Mount Sinai Health System, New York, NY

**Keywords:** surgeon, tricuspid regurgitation, transcatheter edge-to-edge repair, transcatheter tricuspid valve replacement, heart team, imaging

**Figure:**
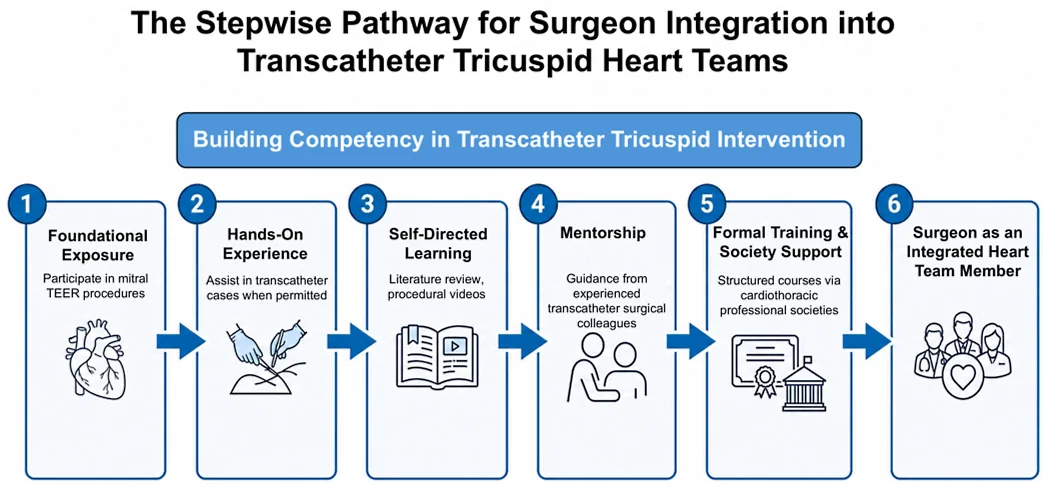
Stepwise pathway for surgeon integration into tricuspid heart teams.

**Central Message**

Integrating cardiac surgeons into tricuspid heart teams via training and participation improves multidisciplinary decision-making, complication management, and long-term care in transcatheter therapy.

See Commentary on page XXX.

Tricuspid regurgitation (TR) has long been considered the neglected valvular disease compared with mitral-valve and aortic-valve disease in terms of attention from clinicians and researchers.^1^ This is alarming, because there are approximately 1.6 million people in the United States with moderate-to-severe or greater TR.^1^ Most of these cases are secondary TR linked to a dilated annulus and right ventricular remodeling, rather than issues with primary leaflet pathology.^1^ Historically, tricuspid-valve surgery has always been rare because of the high risk associated with reoperative and isolated right-sided valve surgery.^1^ This risk is more significant in patients with advanced right ventricular dysfunction, renal and hepatic dysfunction, or a history of previous cardiac surgery.^1^ Recent data from the Society of Thoracic Surgeons database showed that despite improvement in outcomes after isolated tricuspid-valve surgery, morbidities including prolonged mechanical ventilation and length of stay remain.^2^^,^^3^ Transcatheter edge-to-edge repair (TEER) and transcatheter tricuspid valve replacement (TTVR) have therefore significantly transformed the management of TR. These procedures provide undertreated patients with a minimally invasive alternative for those unable to undergo open heart surgery. However, as this therapy continues to evolve, the challenge of maintaining an intact multidisciplinary heart team emerges. Those who have the most experience in dealing with tricuspid anatomy and pathology, cardiac surgeons, find themselves outside the key decision-making and implanting team of transcatheter therapy for TR. Our Invited Expert Opinion focuses on the roots of this problem, the need to reverse it, and possible solutions.

## Transcatheter Tricuspid Interventions: Innovations Driving Rapid Advancement

**Figure 1:**
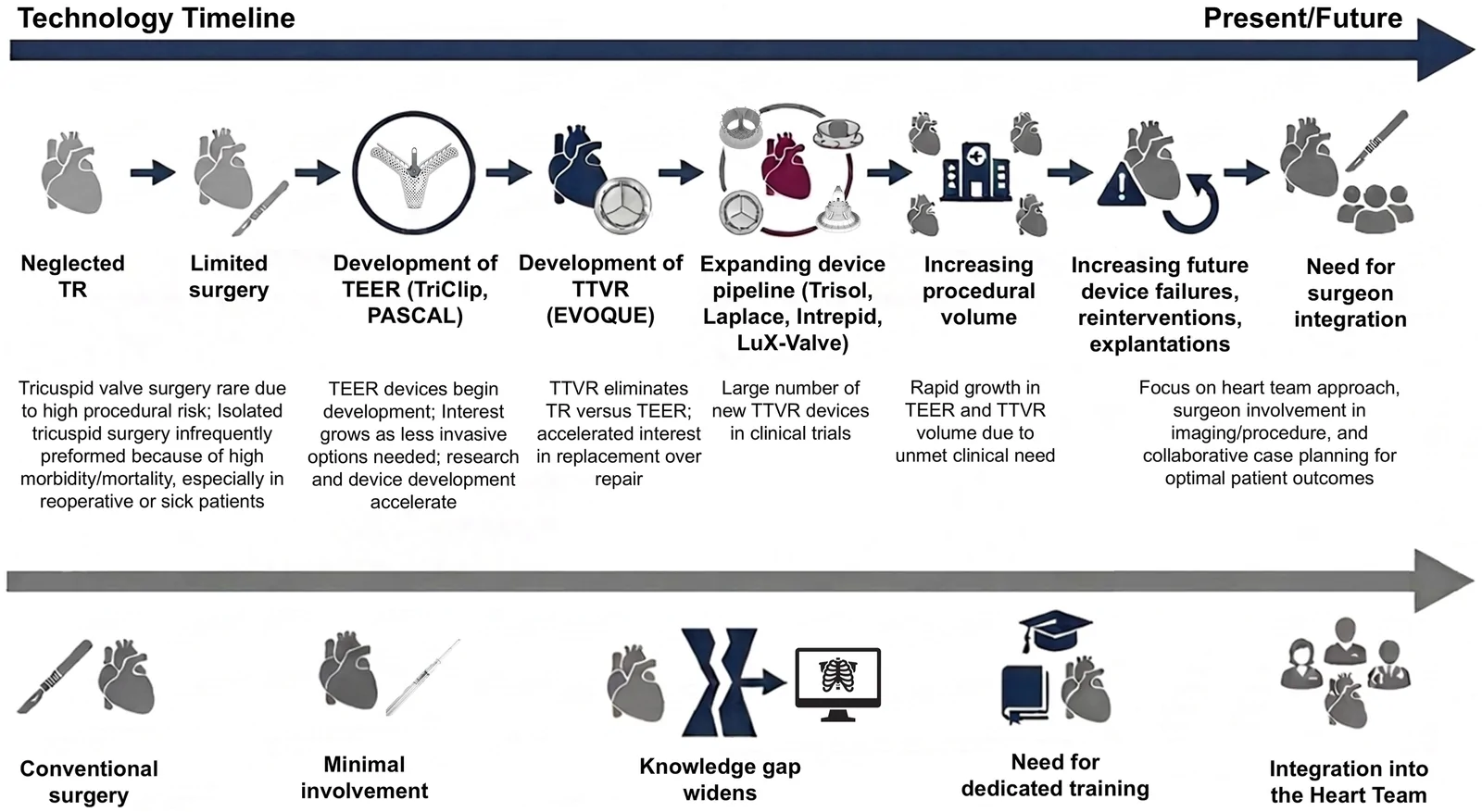
Timeline of evolution of transcatheter tricuspid interventions and evolving role of cardiac surgeons in this context. Development of technology in parallel with changes in the level of surgeons' participation throughout history, emphasizing the importance of surgical involvement in current transcatheter heart teams. *TR*, Tricuspid regurgitation; *TEER*, transcatheter edge-to-edge repair; *TTVR*, transcatheter tricuspid valve replacement.

Transcatheter tricuspid-valve devices have emerged rapidly within the past several years (Figure 1), focusing on 2 major strategies: repair and replacement. TEER devices like the TriClip (Abbott Structural Heart) and PASCAL (Edwards Lifesciences) systems apply the same repair principles used in the treatment of mitral regurgitation to TR.^4^ In parallel, TTVR technology has significantly advanced with the EVOQUE Transcatheter Tricuspid Valve Replacement System (Edwards Lifesciences).^5^ This includes a nitinol self-expanding frame with bovine pericardial leaflets available in 4 sizes and features an intra-annular sealing skirt, multiple ventricular fixation points, transfemoral delivery system, and multiplanar steerable platform.^5^^,^^6^ In addition, a number of new technologies of repair and replacement are currently progressing through clinical trials.^7^ Some of these include the PASCAL Precision system for TEER (Conformité Européenne marked only) and Trisol, Laplace, Intrepid, and LuX-valve systems for TTVR.^7^ The rapid evolution of transcatheter tricuspid-valve therapy demonstrates that it is becoming an integral component of structural heart intervention in tricuspid valve disease.

## Surgical Conversion in TTVR Remains Rare But Challenging

**Figure 2:**
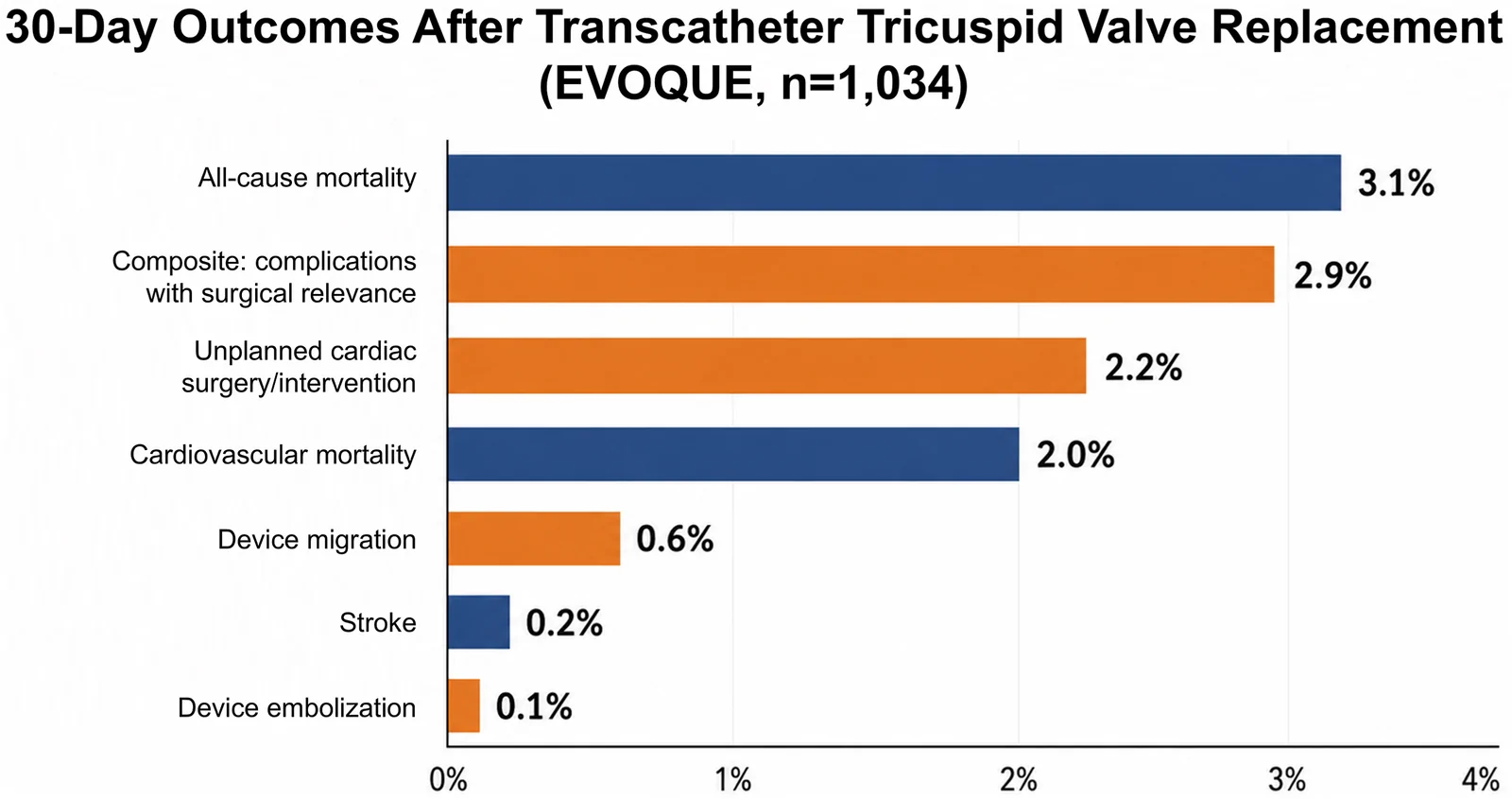
Clinical outcomes and complications after transcatheter tricuspid valve replacement in the real world. Thirty-day clinical event rates after transcatheter tricuspid valve replacement using the EVOQUE system from the Society of Thoracic Surgeons/American College of Cardiology Transcatheter Valve Therapy registry.^9^*Orange bars* illustrate complications that are pertinent to the practice of cardiac surgeons, such as device migration (0.6%), device embolization (0.1%), and unplanned cardiac surgery/intervention (2.2%). The complication composite with clinical relevance to surgery (2.9%) is the cumulative rate for these particular intraprocedural or postprocedural structural events.

Although tricuspid TEER is extremely safe, with surgical intervention being rare, TTVR in the real-world remained at an increased risk over TEER.^8^^,^^9^ A recent analysis of 1034 patients treated with the EVOQUE TTVR system showed that within 30 days, the overall mortality rate was 3.1%, cardiovascular mortality 2.0%, and stroke rate only 0.2% (Figure 2).^9^ This demonstrates that TTVR can be performed with acceptable rates of adverse events in a variety of health care facilities. However, 0.1% of the cases had device embolization, 0.6% had device migration, and 2.2% of the cases had unplanned cardiac surgery or intervention.^9^ In cases of valve misplacement, embolization, and immediate surgery, the surgeon's understanding of the anchoring mechanics and failure mode of the implanted device is crucial. This concern extends beyond just the periprocedural period. As in any other case of valvular prosthesis, some of the patients will eventually require device explantation due to structural valve deterioration, significant paravalvular leakage, or endocarditis. There are reported cases of surgical bailout and transcatheter valve-in-valve implantation of the maldeployed transcatheter tricuspid valve.^10^ Surgeons need to know the device design to be able to manage these complications safely and expeditiously.^10^

## Why Have Surgeons Not Been Actively Involved In Transcatheter Tricuspid Procedures?

**Figure 3:**
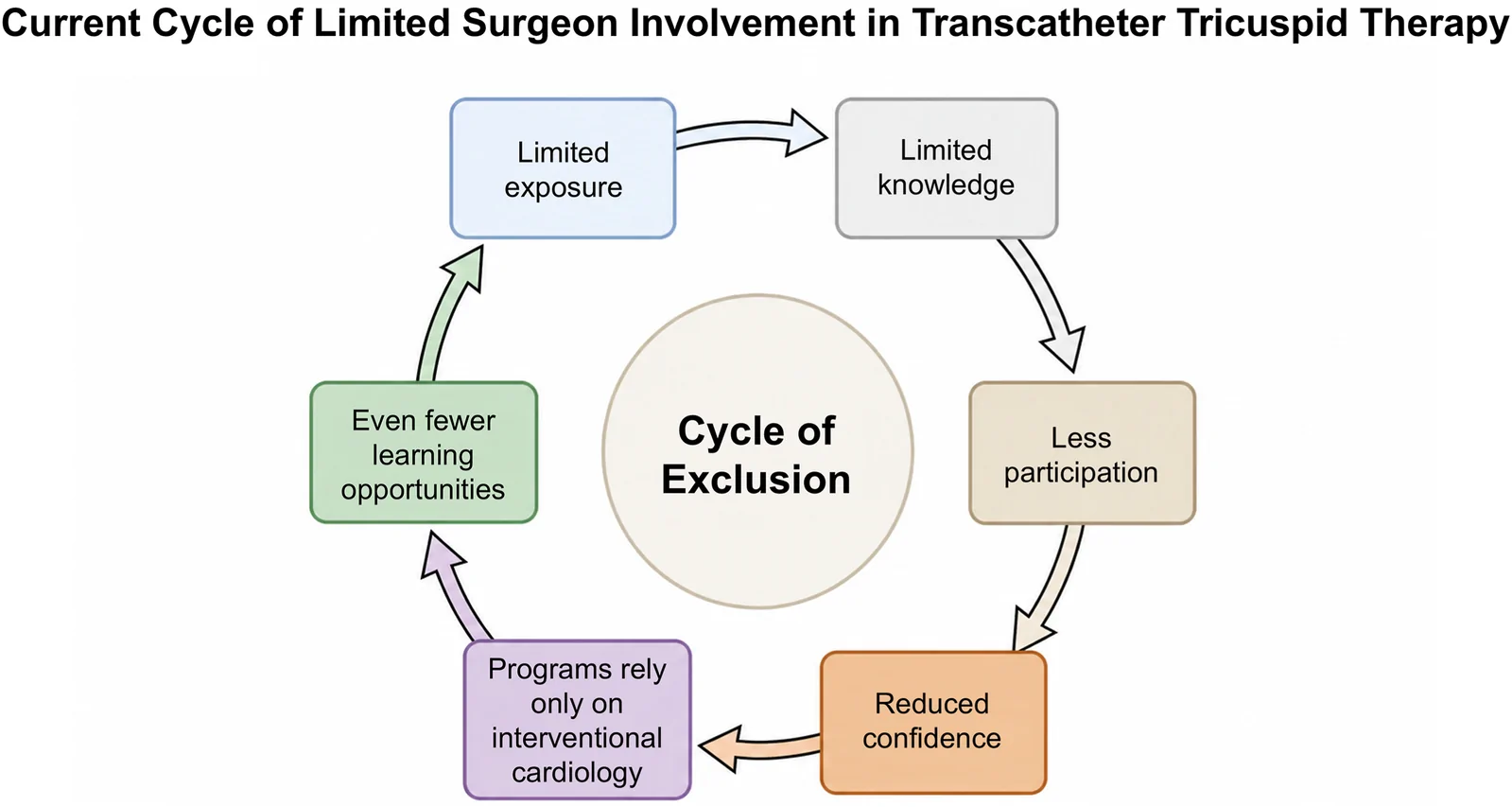
Self-reinforcing cycle of limited cardiac surgeon engagement in transcatheter therapies for the tricuspid valve. Diagram explaining how low exposure and lack of knowledge contribute to low participation and engagement, resulting in the program becoming exclusively interventional cardiology–led.

Currently, there is a relative lack of engagement from cardiac surgeons within transcatheter tricuspid programs (Figure 3).^11^ This stems from a combination of structural and educational barriers. The absence of cardiac surgeons from mitral TEER programs has led to a generation of cardiac surgeons lacking firsthand experience in the basic principles of transcatheter atrioventricular valve repair, imaging guidance, and procedural planning.^12^ In addition, there is a communication barrier between surgeons and cardiologists because of the use of an unfamiliar language of imaging guidance during a procedure. Although surgeons understand transesophageal echocardiography, specific imaging terminology used by structural imagers and implanters, such as real-time multiplanar reconstruction in image guidance and fluoroscopy, becomes foreign to them.^12^ This makes it difficult for surgeons to provide valuable insight to the heart team. Moreover, many surgeons do not grasp fluoroscopic navigation because their training has mostly revolved around direct visualization.^12^ This lack of knowledge limits their ability to contribute to transcatheter tricuspid programs, which in turn creates a cycle that excludes them from these opportunities.^11^ The challenges increase when working with the tricuspid valve rather than the mitral valve. Navigating the tricuspid valve during transcatheter procedures is more difficult because of its thinner leaflets, far field from the transesophageal echocardiography probe, and shadowing from adjacent structures especially prostheses.^7^ Surgeons lacking experience in transcatheter mitral procedures face an additional hurdle compared with their interventional cardiology peers, who have had years of training in structural heart procedures.^12^

## Opportunities For Surgeons To Offer Value To Transcatheter Tricuspid Therapy

Despite these difficulties, there is a strong reason for surgeons to participate in transcatheter tricuspid programs, as evidenced by the recommendations made in the recently published multisociety document.^13^ Surgeons possess extensive anatomical and pathophysiological knowledge of the tricuspid valve, gathered from years of performing surgical repair and replacement.^11^ This expertise can be applied directly during clinical decision-making and the planning of transcatheter procedures, where a solid understanding of pathoanatomy, pathophysiology, and tissue quality would be valuable.^11^ Surgeons can develop skills in interpreting transesophageal and intracardiac echocardiographic images and can relate this information to their existing understanding of pathoanatomy.^12^ With structured training, they can also develop fluoroscopy skills. Once they become proficient in these core skill sets, they can become valuable contributors to heart teams. They can contribute to case selection, identify potential anatomical problems for transcatheter interventions, and provide surgical backup when transcatheter intervention fails or when the patient/anatomy is better suited for surgery.^11^ Programs that integrate surgical expertise into their transcatheter heart teams have reported improved outcomes and greater efficiencies. In addition, occurrences of device failure that require explantation further highlight the importance of involving cardiac surgeons in the process.^10^ With the increasing volume of transcatheter tricuspid procedures, the number of patients facing complications such as device embolization, thrombosis, infection, and device failure is bound to increase.^10^ Without the surgical team being familiar with the specific designs and failure mechanisms of these transcatheter valves, dealing with these problems surgically would become increasingly difficult and risky for the patient. Therefore, involvement of the surgeon at the initial stages of a transcatheter tricuspid program development should be viewed as essential rather than optional.

Clearly, addressing this issue requires a thoughtful, step-by-step strategy. Surgeons should start by participating in mitral TEER procedures as a way to acquire the necessary skills for transcatheter treatment of mitral and tricuspid valve diseases.^12^ They should participate in transcatheter procedures and, when allowed by their institutions and peers, assist in these cases. Observing procedures in real time is a much faster way to build proficiency. In addition, self-education is important in this process. Surgeons should familiarize themselves with the rapidly growing scientific literature on transcatheter tricuspid interventions and review online procedural videos and demonstrations. They should actively participate in industry training courses to become knowledgeable in the available therapies. Moreover, mentorship from surgical colleagues who already possess extensive experience in transcatheter tricuspid procedures should be sought out. Finally, cardiothoracic professional societies should provide training courses to train surgeons in becoming competent in transcatheter procedures and imaging.^12^

## Conclusions

**Figure 4:**
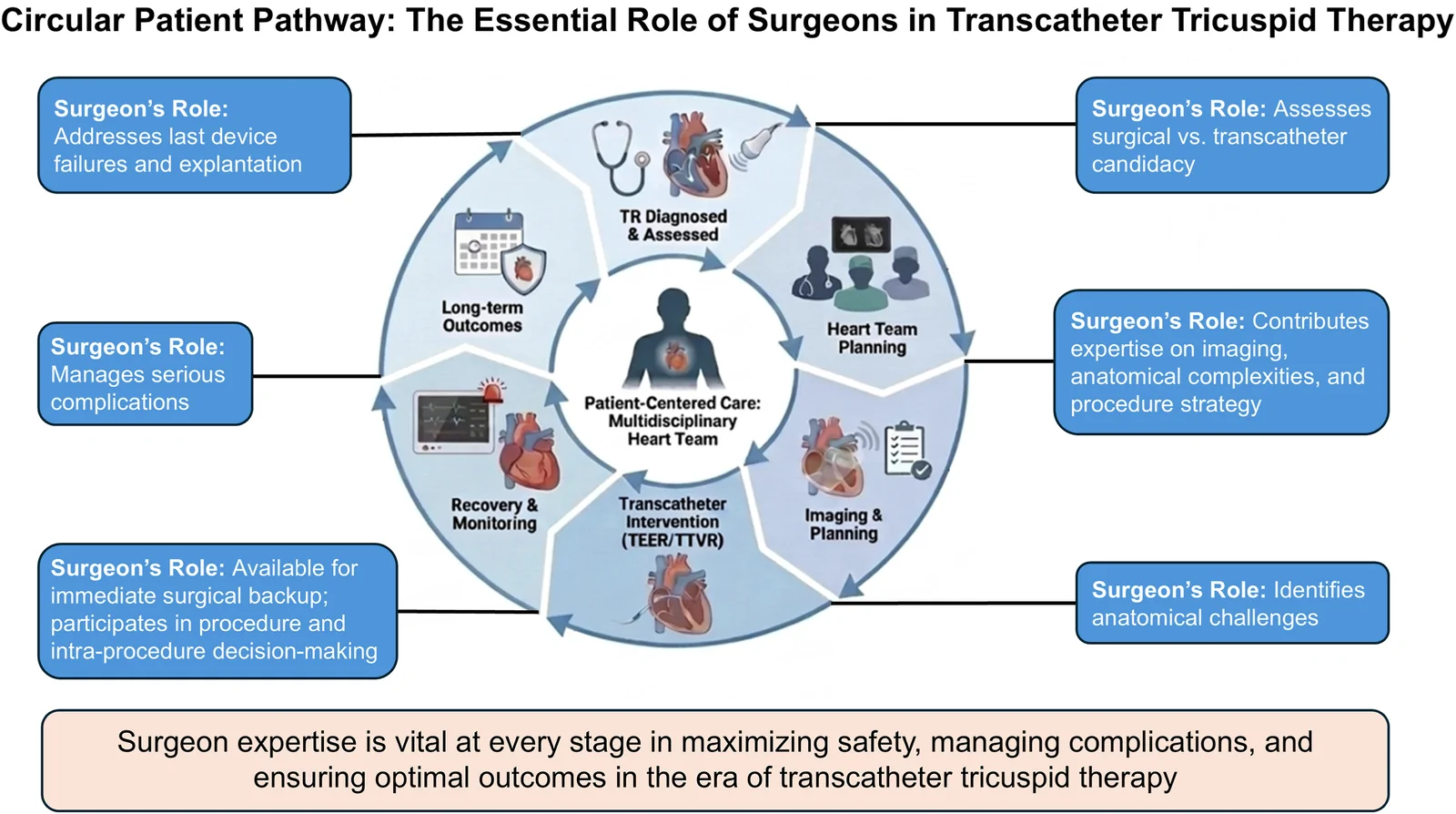
Patient care pathway with emphasis on cardiac surgeon engagement during critical phases. Diagram highlighting the importance of the cardiac surgeon involvement throughout diagnosis, planning, procedure, complications, and follow-up in patients with tricuspid-valve disease. *TR*, Tricuspid regurgitation; *TEER*, transcatheter edge-to-edge repair; *TTVR*, transcatheter tricuspid valve replacement.

The field of transcatheter tricuspid valve treatment is growing rapidly on the basis of a diverse device pipeline and promising real-world results.^7^ However, waning interest, the lack of transcatheter mitral-valve procedural experience, unfamiliarity with imaging terminology, and limited dedicated training opportunities have steered surgeons away.^12^ With the expected increase in frequency of device explantations associated with transcatheter tricuspid device failure, the lack of surgical expertise within heart teams may not serve patients in their best interest.^11^ Surgeons have years of anatomical and pathophysiologic experience working with tricuspid valves. By engaging in heart team decision-making and case planning, and learning and participating in transcatheter procedures through dedicated training or mentorship with experienced colleagues, surgeons will be able to contribute significantly to these procedures and help the heart team in a meaningful way (Figure 4).

## Conflict of Interest Statement

Dr Tang has received speaker's honoraria and served as a physician proctor, consultant, advisory board member, TAVR publications committee member, RESTORE study steering and screening committee member, APOLLO trial screening committee member, and IMPACT MR steering committee member for Medtronic; has received speaker's honoraria and served as a physician proctor, consultant, advisory board member, ENVISION trial screening committee member, and TRILUMINATE trial anatomic eligibility and publications committee member for Abbott Structural Heart; and has served as an advisory board member for Boston Scientific and Pi-Cardia and a consultant for Philips, Edwards Lifesciences, Peija Medical, and Shenqi Medical Technology. All other authors reported no conflicts of interest.

The *Journal* policy requires editors and reviewers to disclose conflicts of interest and to decline handling or reviewing manuscripts for which they may have a conflict of interest. The editors and reviewers of this article have no conflicts of interest.
